# Evaluation of Cone-Beam Computed Tomography Scans to Develop a Staging Method of External Carotid Artery Calcification

**DOI:** 10.3390/jcm13113189

**Published:** 2024-05-29

**Authors:** Varsha Kadyan, Anusha Vaddi, Archna Nagpal, Marco R. Molina, Alan G. Lurie, Aditya Tadinada

**Affiliations:** 1 Division of Oral and Maxillofacial Radiology, University of Connecticut School of Dental Medicine, 263 Farmington Avenue, Farmington, CT 06030, USA; varshakadyan15@gmail.com (V.K.); arcsharma@uchc.edu (A.N.); lurie@uchc.edu (A.G.L.); tadinada@uchc.edu (A.T.); 2 Oral and Maxillofacial Radiology, Department of Oral Diagnostic Sciences, Virginia Commonwealth University School of Dentistry, Richmond, VA 23298, USA; 3 Department of Diagnostic Imaging, University of Connecticut School of Medicine, 263 Farmington Avenue, Farmington, CT 06030, USA; molina@uchc.edu

**Keywords:** carotid artery diseases, atherosclerosis, cone-beam computed tomography, classification, carotid artery, external

## Abstract

**Background:** The objective of this study was to develop a practical staging method for reporting external carotid artery calcifications (ECACs) using cone-beam computed tomography (CBCT) imaging, specifically to standardize reporting for oral and maxillofacial radiologists. **Methods:** This retrospective study evaluated 489 CBCT scans for the presence of ECACs. Two calibrated evaluators assessed the scans in all three orthogonal planes, using the axial plane to develop the staging system. Calcifications were graded on a scale from 0 to 5. **Results:** ECACs were found in 170 out of 489 scans (34.7%). There was a statistically significant increase in ECAC distribution with age progression. The prevalence of ECACs was similar between genders. Grade 1 calcifications were most common in the 51–60 age group, Grade 2 in the 61–70 and 71–80 groups, and Grades 3 and 4 in the 81–90 group. No Grade 5 calcifications were observed in any age group. The inter-rater reliability showed an excellent correlation in the identification and grading of ECACs. **Conclusions:** The proposed grading system enables oral and maxillofacial radiologists to quantitatively report ECACs, facilitating timely referrals to physicians for further evaluation and early intervention, thereby potentially reducing the risk of cardiovascular events

## 1. Introduction

The carotid arteries supply oxygen-rich blood to the head, neck region, and the brain. These arteries bifurcate into the external and internal carotid arteries at the C3–C4 cervical vertebrae level. The accumulation of plaque, cholesterol, fats, and other substances in the carotid artery is known as carotid artery atherosclerosis, which causes narrowing of the blood vessel wall, leading to stenosis. Atherosclerosis occurs in response to a physical or metabolic injury to the vessel wall. Inflammatory response to the damage manifests as deposition of fatty streaks, leading to plaque formation. This plaque reduces the arterial luminal diameter. Eventually, the plaque might calcify, further increasing the severity of stenosis. Stenosis increases the risk of stroke, and studies have shown a correlation between the severity of stenosis and the risk of stroke [1]. Carotid arterial atherosclerotic disease accounts for 20% of all ischemic strokes and transient ischemic attacks (TIAs) [2,3]. The bifurcation area of the common carotid artery is more predisposed to atherosclerosis and subsequent calcification. The vessel’s anatomy, diameter, and low wall shear stress at arterial bifurcations enhance plaque formation. In addition, the disturbance in blood flow pattern at bifurcation coupled with low wall shear stress promotes endothelial dysfunction, leading to atherosclerosis formation and subsequent calcification [4].

Doppler ultrasound (US) is used as the first-line imaging in asymptomatic patients with suspected calcification or a carotid bruit. Doppler US provides information regarding plaque stenosis and flow velocity. Angiography is a traditional and definitive method for the evaluation of the vascular lumen. It provides excellent resolution but does not show the vascular wall and therefore is insensitive for early detection and estimation of lesion volume. Intravascular ultrasound provides cross-sectional images that show the vascular wall, including details that provide insight into lesion composition as well as lumen contour. Computed tomography (CT) angiography or magnetic resonance angiography (MRA) are recommended for therapeutic intervention [5]. MR angiography, due to its non-invasive nature, promises to supplant invasive angiography for the study of major vessels such as the aorta and carotid arteries [6,7].

Extracranial carotid artery calcifications (ECACs) are one of the most common incidental findings on panoramic radiographs and cone-beam computed tomography (CBCT) scans. Studies showed a broad spectrum of prevalence rates for carotid artery calcifications in dental images, ranging from 2% to as high as 31.57% [3,8,9]. Given that vascular calcification indicates advanced atherosclerosis, moderate to severe carotid calcifications warrant referral to the patient’s physician [9,10,11]. Consequently, early identification of carotid calcifications may play a crucial role in identifying undiagnosed coronary artery disease and promptly referring these patients for further evaluation.

Dentists and oral and maxillofacial radiologists (OMFRs) currently face a challenge due to the absence of a reliable radiographic staging system or classifications for ECACs detected in CBCT scans. Accurate classification of these calcifications is crucial for effectively categorizing patients and referring them for further assessment and timely treatment. The majority of existing classifications for carotid artery calcifications are based on CT, magnetic resonance imaging (MRI), or US imaging [12,13,14]. Hence, the study aimed to develop a practical staging method for reporting ECACs specific to CBCT imaging to standardize reporting practices for OMFRs.

## 2. Materials and Method

In this retrospective CBCT study, 500 de-identified CBCT scans were retrieved from oral radiology archives, at the UConn School of Dental Medicine. The scans included were acquired for multiple implant placements in both jaws, adult orthodontics, evaluation of jaw pathologies, and evaluation of temporomandibular joints. The CBCT scans with motion or metallic artifacts, as well as scans that did not capture the C3–C4 region during the acquisition, were excluded from the study.

### 2.1. Acquisition and Scan Parameters

The scans were acquired using a CBCT scanner, 3D Accuitomo (J Morita Corp, Kyoto, Japan). The field of view (FOV) of the scans included was 140 × 100 mm and 170 × 120 mm. The machine operates with predefined protocols (low, medium, high dose). For adult and elderly patients (aged 30–90 years), the medium-dose protocol is typically employed. However, in cases of extreme variation in patient density, the protocol is adjusted to either low or high doses as appropriate. In this study group, we used the medium-dose protocol for all patients.

The acquisition parameters for the medium-dose protocol scan were 90 kVp, 7 mA, and 17.5 s, with a voxel size of 250 µm.

### 2.2. Scan Evaluation

Two oral radiology residents in training independently assessed the scans. Their selection was based on their rigorous academic training and hands-on experience in oral radiology, which provided them with the necessary skills to accurately evaluate CBCT images. They were blinded to all patient records such as medical and dental history. However, they had access to the age and gender of each patient. Using a CBCT reconstruction program, Invivo version 6.0 (Anatomage, Santa Clara, CA, USA), the scans were meticulously assessed for ECACs. The credibility of Invivo version 6.0 software is confirmed by data from prior studies demonstrating its efficient visualization of anatomy and pathological structures for diagnostic purposes [15].

The calcifications were studied and evaluated in all three orthogonal planes, but the axial plane was used to develop the staging, using the soft-tissue window of the Invivo software program to best visualize vessel anatomy and any possible calcifications. The slice thickness was set to 0.1 mm. All the observations were performed on an LCD monitor with a 1920 × 1080 pixel screen resolution and adjustable screen brightness.

The ECACs were determined as calcifications in the cervical soft tissues lateral to the anterior tubercle of the transverse processes and posterolateral to the greater cornu of the hyoid bone and pharyngeal airway [7]. The calcifications were categorized as left, right, and uni/bilateral. In the case of bilateral ECACs, both the right- and the left-sided calcifications were graded individually, and the higher grade was considered the final grade for ECACs.

**Figure 1:**
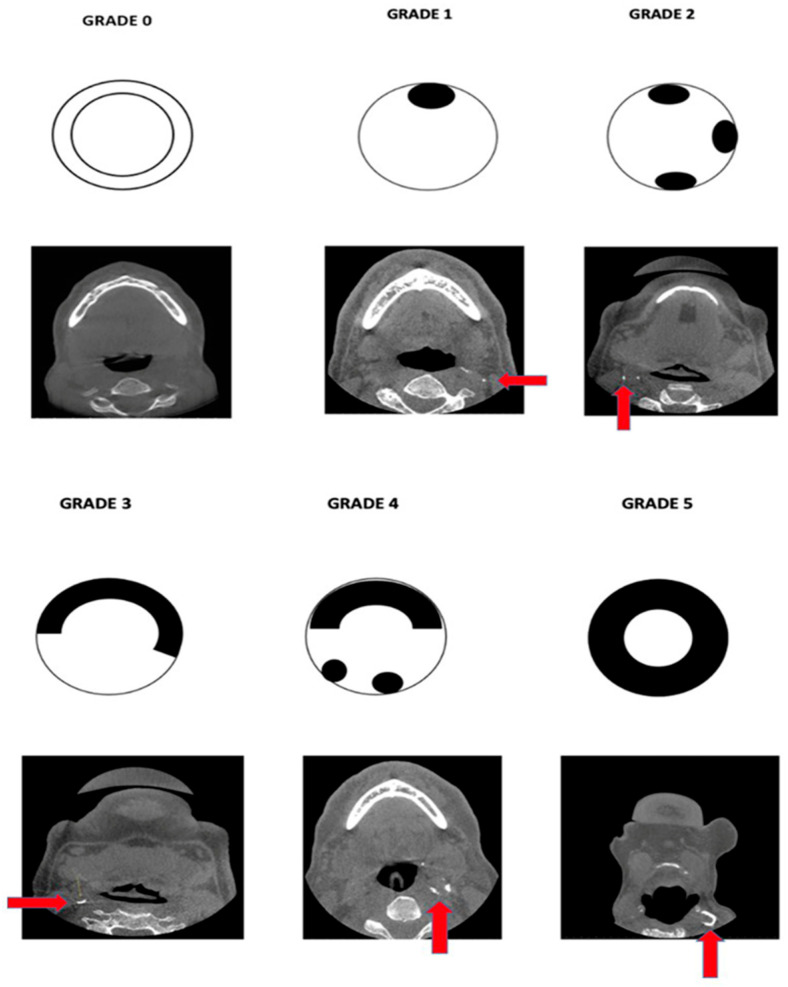
Pictorial representation of the proposed classification scheme along with the corresponding axial-section CBCT images.

**Figure 2:**
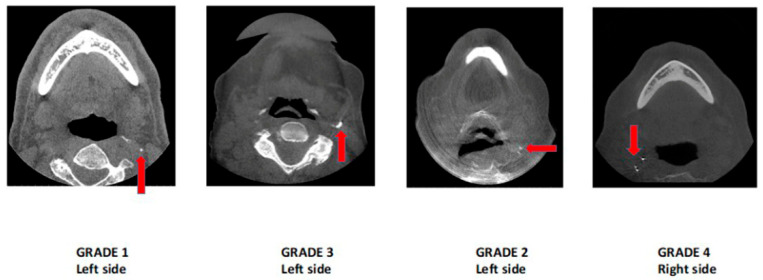
Axial-section CBCT images demonstrating unilateral ECACs according to the proposed grading scheme.

**Figure 3:**
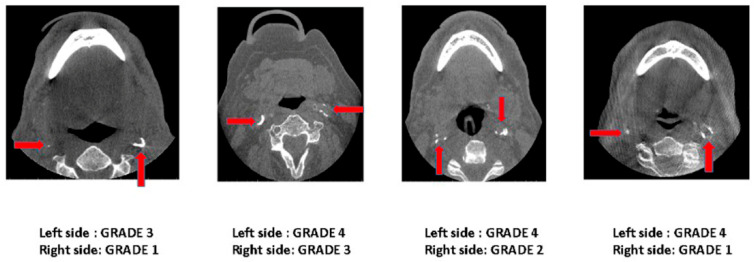
Axial-section CBCT images demonstrating bilateral ECACs according to the proposed grading scheme.

The staging system of ECACs was proposed based on the extent and continuity of the calcified plaque in the internal lumen of the artery at the bifurcation area; the ECACs were allocated into six categories (Table 1, Figure 1). This classification was used for both unilateral and bilateral calcifications (Figure 2 and Figure 3).

Before the study began, the evaluators underwent calibration and convened for a consensus meeting with a board-certified oral radiologist (AT), who possesses extensive expertise in the field, to establish agreement on the radiographic appearance and classification. In cases of disagreement, the evaluators engaged in discussions to resolve the disagreement. For example, distinguishing Grade 1 calcifications from triticeous cartilage calcifications posed a challenge in the study. Triticeous cartilage manifested as a single, oval-shaped opacity located posteromedial to the greater cornu of the hyoid bone, while Grade 1 carotid artery calcifications appeared posterolateral to the greater cornu and the pharyngeal space.

In the first session, the first 50 scans were graded together, then all the remaining scans were graded separately and independently by both raters. Both the raters used Invivo 6 reconstruction programs for image analysis. The raters used multiple planar reformation (MPR), and they were allowed to change the contrast and density of the images. Final grading was performed by using the axial section. The findings were recorded in a pre-designed study proforma.

### 2.3. Statistical Analysis

Descriptive statistics were performed using IBM SPSS Statistics 19 (IBM Corporation, Armonk, New York, NY, USA). The calcifications were graded on a scale of 0–5. In addition, the unilateral and bilateral distribution of calcifications was analyzed. The ECACs were studied based on age and gender distribution. The percentage distribution was calculated, and the chi-square test was used to analyze the data. The inter-rater reliability was analyzed by using Krippendorff’s Alpha test.

## 3. Results

A total of 500 de-identified CBCT scans were reviewed, with 11 scans excluded due to artifacts and the absence of the C3–C4 area in the scan volume. The final analysis included 489 CBCT scans, where ECACs were observed in 170 (34.7%) scans. A chi-square test indicated highly significant differences in ECAC distribution among the studied age groups. The highest percentage of calcifications (33.9%) was found in the 61–70 age group. The calcifications were not detected in the 31–40 age group. There was a minimum number of calcifications in the 41–50 age group. The percentage distribution of ECACs increased with age progression. The prevalence of ECACs increased with age, with unilateral ECACs most common in the 61–70 age group and bilateral ECACs in the 71–80 age group (Table 2).

Out of the 489 CBCT scans, 57.5% were from females and 42.5% from males, with ECACs observed in 74 males and 96 females without a significant gender-based difference (Table 3).

The ECACs were graded based on the proposed grading schematic (Table 1, Figure 1). The distribution of Grade 1 ECACs was higher in the 51–60 age group, Grade 2 in the 61–70 and 71–80 age groups, and Grades 3 and 4 in the 81–90 age group. Grade 5 calcifications were not found in any age group. A significant correlation was found between age and the severity of calcifications, with older age groups exhibiting higher grades of calcification (Table 4).

The inter-rater reliability was analyzed by using Krippendorff’s Alpha. The results of the test ranged from 0.94 to 1, demonstrating excellent correlation in the overall identification and grading of ECACs (Table 5).

## 4. Discussion

Atherosclerosis of the extracranial carotid arteries is an established cause of stroke [16]. The mechanism of calcification in atherosclerosis is still uncertain. Calcification in atherosclerosis is considered a regressive alteration of the plaque. Some authors propose it as a regulated process based on histological resemblance to trabecular bone. Histological studies demonstrated mild calcifications are associated with soft plaque and medium-to-severe calcifications are associated with hard plaques [17].

The current study has proposed a staging system for ECACs in CBCT imaging. CBCT scans with a large field of view (FOV) often depict both extra- and intracranial carotid artery calcifications, and significant associations between both have been well established in the literature [9]. To minimize the radiation dose, the FOV is often reduced in certain dental examinations that tend to show ECACs more often than the intracranial vessels. These vascular calcifications are often carefully assessed by OMFRs on CBCT scans. It may be valuable to identify, stage, and report the presence of carotid calcifications in CBCT scans for early intervention and to adhere to legal liabilities and implications [18]. This is because stenosis or advanced calcifications in the cervical area, especially near the bifurcation area, pose a significant risk for cardiovascular events or cerebral ischemia [19]. The calcification in the bifurcation area can serve as a useful marker for the presence and location of a carotid plaque. Timely referral of these patients can be a lifesaver or can result in a better prognosis of cardiac disease.

There are different staging systems for grading carotid calcifications based on imaging. Doris et al. reported extracranial carotid artery calcification based on plain-film radiography. Grading was based on a system of 0 through 10, with 0 being no calcification, with progression from single and multiple punctate calcifications to a linear plaque on one side of the artery, on two opposite sides, and continuous across both sides of the artery. In Grade 10, the calcification was completely circumferential [20]. Phyo et al. classified carotid calcifications based on computed tomography. The authors described calcifications based on the size as micro (calcification less than 2 mm) and macro calcifications (calcification greater than 2 mm) and mixed, comprising both micro and macro calcifications [21].

With the advent of CBCT, the visualization of these calcifications has become more reliable in dental imaging. In the literature, there are no reliable or clinically practical staging systems or classifications for reporting ECACs in CBCT images. The proposed staging method for calcification in extracranial carotid arteries is a visual score method and is adapted from Babiarz’s [12] and Woodcock’s [13] grading of ICA. Woodcock [13] et al.’s classification is a visual scoring method to characterize the ICA siphon calcifications. The authors graded calcifications as absent, mild (thin, discontinuous), moderate (thin, continuous, or thick, discontinuous), or severe (thick, continuous) on axial CT. Woodcock’s method demonstrated excellent inter- and intra-rater agreements and was more straightforward and less time-consuming than some of the other visual methods. Similarly, our proposed classification is clear and comprehensive and can be standardized for referral and effective communication between OMFRs and medical professionals.

In the present study, there was a significant difference in the distribution of ECACs based on age of patients. There was a rise in the percentage of calcifications with increasing age. In the 81–90 age group, 83.3% of patients had calcifications. This agrees with previous studies [9,19]. Ertas and Sisman et al. reported a statistically significant increase in ECACs in the 40–49 age group to the 70–79 age group [22]. Johansson et al. in their panoramic image-based study found that the mean age for persons with calcification in the area of the carotid arteries was higher compared to individuals without calcification in the area of the carotid arteries [23]. The possible reason could be due to an increase in the tortuosity of the arteries with increasing age. With advancing age, the intima becomes thickened and fibrosed, and the medial smooth muscle and elastic fibers are partly replaced by collagen. Hence, they become more susceptible to calcification [24]. Mutalik and Tadinada suggested including patients with a lesser risk of carotid calcification, those below the age of 40 years, to find the true prevalence of vascular calcifications in the younger age group [9]. In the present study, the 31–40 age group was included. However, no calcifications were reported in that age group. A possible explanation could be that in young patients around 30 years old, the external carotid arteries might be straight. The tortuosity of these arteries eventually increases with age [24].

There was no significant difference in the distribution of calcifications based on gender. This finding correlates with the study by Ertas and Sisman [22]. Johansson et al. [23] in their study reported a higher rate of plaque formation and calcification in males. However, such correlation was not found in the current study. The previous literature showed a correlation between smoking and atherosclerotic plaque; hence, males are more likely to develop plaques. On the other hand, postmenopausal females are more prone to the formation of plaques [21].

There was a significant difference in the pattern of calcifications with increasing age. Single and multiple specks of calcification were noted in those aged 41 to 50 (Grade 1, 2). In addition to the specks, a semicircular band was noted in the 51–60 age group (Grades 1–3), and all four patterns (Grades 1–4) were present in the 61–90 age group. Culebras et al. strongly suggested a direct correlation between advancing age and the deposition of calcium in the carotid arteries [25].

Circumferential calcifications, which represent the most advanced stage of the disease, were not noted in any patients. In Babiarz’s classification [12], the amount of calcification equates with the degree of plaque burden in the coronary arteries, emphasizing the association of advanced lesions with a diffuse circumferential pattern of calcium distribution as opposed to point-like presence. Culebras et al. in their CT study of cervical carotid calcification mentioned that the thickness of the atherosclerotic plaque in the walls of the artery varies. The plaques are usually thickest near the midpoint of the lateral carotid sinus wall, opposing the high shear stress point. The shallow plaques tend to be more heavily calcified than thick ones, and the thickest segment of a plaque is not usually calcified, whereas the adjacent thin rim of atherosclerosis might be calcified [25].

A significant limitation of CBCT scans is their inability to demonstrate soft-tissue occlusive components. As a result, the study cannot fully assess the extent of plaque, particularly in terms of non-calcified components. The high inter-rater reliability observed in the current study indicates that the scale and staging system can be easily replicated for use in large-scale epidemiological studies. However, it is important to note that the study’s limitation lies in evaluating only a single calcification site. Another limitation of the study is the lack of angiograms or Doppler US to confirm the presence, extent, or severity of vessel stenosis due to calcification. Additional potential limitations of the study include the sample size and the demographics of the studied group. Future research should focus on expanding the sample size in diverse populations and assessing multiple calcification sites. Incorporating a quantitative calcium score could enhance the study’s methodology. Furthermore, considering higher-resolution scans to measure vessel diameter may be a valuable consideration for future investigations.

Although precise diagnosis for treatment planning necessitates other imaging techniques, CBCT scans are extensively employed in elderly patients for dental implant placement and pathology assessment [26]. OMFRs encounter ECACs on a routine basis, emphasizing the importance of assessing and reporting these conditions for early detection. Barghan et al. [27] reported incidental extracranial carotid artery calcifications in 10.41% of their study sample of 400 scans. Similarly, Most et al. [8] found a prevalence of carotid artery calcifications in 27.8% of their study sample, which included 169 patients identified through various dental imaging methods. Due to the high incidence rate, it is not feasible to refer all the patients with even minimal calcifications. For mild calcifications (Grade 1 and 2), correlation with the patient’s history and risk factors such as age, smoking, and underlying conditions is recommended before considering referral. Moderate-to-severe carotid calcifications detected in CBCT warrant prompt referral to the patient’s physician for further evaluation and management, as these represent mature calcific deposits with a higher likelihood of causing stenosis (Grades 3, 4, and 5) [9,28,29]. Currently, there are no guidelines regarding the referral criteria for external carotid artery calcifications observed in dental imaging, highlighting the imperative for future research to establish such guidelines.

## 5. Conclusions

The suggested grading system could help OMFRs quantitatively report ECACs, facilitating patient referrals for further assessment and early intervention to prevent cardiovascular risks. This study introduces a straightforward scoring system with strong inter-operator reliability for evaluating ECACs. Standardizing severity staging simplifies communication with referring physicians, enabling proper condition triage. While improvements are possible, the scoring system addresses commonly encountered scenarios. Simple, visual scoring systems that do not rely on additional software or calculations tend to encourage broader participation, leading to larger epidemiological studies.

## Figures and Tables

**Table 1:** Proposed grading system for ECACs.

Grade	Description
Grade 0	No calcified plaque was present.
Grade 1	Presence of a single speck of calcified plaque.
Grade 2	Presence of more than one/multiple discontinuous specks of calcifications.
Grade 3	One continuous semi-lunar band of calcification, covering at least half of the arterial lumen.
Grade 4	One continuous semi-lunar band of calcification, covering at least half of the arterial lumen, along with one or more discontinuous calcified specks (Grade 2 + Grade 3).
Grade 5	Circumferential coverage of the arterial lumen with calcified plaque.

**Table 2:** Distribution and percentage of ECACs in different age groups.

Age Group	Total No. of Patients in the Age Group	No Calcification	ECAC Left	ECAC Right	Bilateral	Percentage of ECACs
31–40 years	16	16 (100%)	0 (0%)	0 (0%)	0 (0%)	0%
41–50 years	43	38 (88.4%)	3 (7%)	2 (4.7%)	0 (0%)	11.6%
51–60 years	146	111 (76%)	7 (4.8%)	10 (6.8%)	18 (12.3%)	23.9%
61–70 years	166	105 (63.3%)	18 (10.8%)	27 (16.3%)	16 (9.6%)	36.7%
71–80 years	106	47 (44.3%)	15 (14.2%)	13 (12.3%)	31 (29.2%)	55.6%
81–90 years	12	2 (16.7%)	2 (16.7%)	0 (0%)	8 (66.7%)	83.3%
Total	489	319 (65.2%)	45 (9.2%)	52 (10.6%)	73 (14.9%)	34.7%

The *p*-value for the differences in ECAC distribution across different age groups is <0.0001 (highly significant).

**Table 3:** Gender-based distribution and percentage of patients with and without ECACs.

Gender	Total No. of Patients	No Calcification	ECAC Left	ECAC Right	Bilateral	Total No. of ECACs	Percentage of ECACs
Males	208	134 (64.4%)	20 (9.6%)	22 (10.6%)	32 (15.4%)	74	35.6%
Females	281	185 (65.8%)	25 (8.9%)	30 (10.7%)	41 (14.6%)	96	34.2%
Total	489	319 (65.2%)	45 (9.2%)	52 (10.6%)	73 (14.9%)	170	34.7%

The *p*-value for the difference in the distribution of ECACs between males and females is 0.98 (not significant).

**Table 4:** Age-based distribution of calcification grades according to the proposed classification system.

Age Group	Total No. of Patients in the Age Group	Grade 0	Grade 1	Grade 2	Grade 3	Grade 4	Grade 5
31–40 years	16 100%	16 100%	0 0%	0 0%	0 0%	0 0%	0 0%
41–50 years	43 100%	38 88.4%	4 9.3%	1 2.3%	0 0%	0 0%	0 0%
51–60 years	146 100%	108 73.97%	14 9.58%	16 10.95%	8 5.47%	0 0%	0 0%
61–70 years	166 100%	101 60.84%	15 9.03%	30 18.07%	18 10.84%	2 1.2%	0 0%
71–80 years	106 100%	39 36.79%	7 6.6%	27 25.47%	20 18.86%	13 12.26%	0 0%
81–90 years	12 100%	1 8.33%	0 0%	2 16.6%	4 33.3%	5 41.6%	0 0%
Total	489 100%	303 61.96%	40 8.17%	76 15.54%	50 10.22%	20 4.08%	0 0%

The *p*-value for the distribution of calcifications across different age groups is <0.001 (highly significant).

**Table 5:** Inter-rater reliability for identifying and grading ECACs.

Category	Inter-Rater Reliability (Krippendorff’s Alpha) Score
Overall ECA identification	1
Overall grading of ECA calcification	0.978
Subjects in Grade 1	0.945
Subjects in Grade 2	0.965
Subjects in Grade 3	1
Subjects in Grade 4	1
Subjects in Grade 5	1

## Data Availability

Data are unavailable due to privacy or ethical restrictions.
